# The Moroccan Knee Osteoarthritis Outcome Score (KOOS)-Child Scale: Translation, Cultural Adaptation, and Validation

**DOI:** 10.7759/cureus.49832

**Published:** 2023-12-02

**Authors:** Ibtissam El Harch, Nabil Chettahi, Soumaya Benmaamar, Abderahim Kamli, Noura Qarmiche, Nada Otmani, Nabil Tachfouti, Mohamed Berraho, My Abderrahmane Afifi, Samira EL Fakir

**Affiliations:** 1 Laboratory of Epidemiology, Clinical Research and Community Health, Faculty of Medicine and Pharmacy, Sidi Mohamed Ben Abdellah University, Fez, MAR; 2 Department of Pediatric and Orthopedic Surgery, Hassan II University Hospital, Fez, MAR

**Keywords:** morocco, cultural adaptation, questionnaire development and validation, koos-child scale, moroccan koos-child scale

## Abstract

Objective

The objective of this study was to perform a cross-cultural validation and adaptation of the Moroccan Dialectal Arabic version of the Knee Osteoarthritis Outcome Score (KOOS)-Child scale.

Methodology

Two groups of participants were recruited: a group of children affected by knee problems and another group serving as a control, free of any knee pathology. Participants were asked to complete the KOOS-Child scale twice with a minimum interval of 15 days.

Results

This study included 130 patients aged 9.82 ± 3.16 years, comprising 88 (67.7%) patients with knee problems and 42 (32.3%) controls. The baseline comparison showed no statistically significant difference between the two groups. The KOOS-Child scale was translated into Moroccan Dialectal Arabic without encountering difficulties in the translation and cross-cultural adaptation process. It proved practical, reliable, and suitable for assessing problems that children and adolescents with knee disorders may encounter. The scale exhibited good content validity and test-retest reliability.

The Moroccan scale also demonstrated excellent internal consistency, except for the symptoms subscale. Confirmatory factor analysis indicated that the structure of the Moroccan version of the KOOS-Child scale was acceptable.

Conclusions

The Moroccan KOOS-Child scale exhibited good acceptability, reliability, discriminative capacity, and overall good internal consistency, with the exception of the symptoms subscale.

## Introduction

The knee joint is one of the most commonly injured sites in children and adolescents [1,2]. These injuries are predominantly caused by trauma [1,3,4], but they can also be secondary to infectious, tumor, or malformative pathologies [5].

Pain is the main symptom of knee injuries and can result from all types of major or minor injuries [6]. However, limited data are available on pain in children and adolescents [7-10]. Several studies have shown that the impact of this pain can extend beyond normal life, affecting the participation of this population in their school, social, and sporting activities. In some cases, it may even lead to the need for medical or medicosurgical care, with all the associated costs of care [11-14]. The impact of pain in childhood extends beyond this life stage, as emerging evidence suggests that children, particularly adolescents who report enduring pain, face an elevated risk of developing chronic pain as adults [15-17].

Pain communication is less clear in children and adolescents, with causal factors ranging from identifiable to very diffuse [18]. Measuring their health-related quality of life (HRQoL) allows us to gain insight into their subjective pain experiences and how pain impacts various dimensions of their lives [19].

The Knee Osteoarthritis Outcome Score (KOOS) is an originally self-administered questionnaire designed for adults with knee-related dysfunctions [20]. It was used to assess children with knee problems due to the absence of specific pediatric measurement scales. It was not until 2012 that a group of researchers decided to investigate the use of the adult KOOS on children [21]. Their findings revealed a lack of understanding among certain children. To address this, a modified version of the original KOOS, better suited for children, was developed, known as the KOOS-Child. Several adjustments were made in various aspects, including general instructions, language (specifically regarding terminology and grammar), item and response formats, mapping, and layout, all aimed at presenting transitions between questions related to pain or difficulty more clearly. Children's suggestions were actively used to modify the items, and additional questions were incorporated, covering topics such as maintaining balance, participation in sports, school activities, and social engagement [21].

After reducing the items to 39 and dividing them into five subscales, the final KOOS-Child was studied in 115 children aged 7-16 years with knee disorders. The questionnaire proved to have good validity, reliability, and responsiveness, except for internal consistency in the *symptoms* subscale. This inconsistency was explained by the high variability of symptoms that patients experienced due to the diversity of their knee problems [22].

Currently, the KOOS-Child serves as a child-friendly assessment tool in pediatric studies, encompassing a broad spectrum of knee conditions in children. Despite its availability in various versions, there is currently no Moroccan version available.

The main objective of this work is to conduct cross-cultural validation and adaptation of the Moroccan Dialectal Arabic version of the KOOS-Child scale.

## Materials and methods

KOOS-Child scale

The KOOS-Child [23] scale is a patient-reported outcome measure that covers five subscales scored separately: pain (eight items), symptoms (seven items), difficulty during activities of daily living (ADL) (11 items), sport and play (seven items), and quality of life (QOL; six items). Each question is accompanied by standardized answer options presented in five boxes, and respondents assign a score from 0 to 4 to each item, with 0 indicating no problem.

Cross‑cultural adaptation procedure

The cross-cultural adaptation was carried out as follows: First, the questionnaire was independently translated into Moroccan Arabic dialect by two native Moroccan translators with excellent certified knowledge in English. Second, during a meeting, a group of experts obtained a preliminary Moroccan version based on the two independent translations. Third, this preliminary version was back-translated into English by two professional translators with no knowledge of the initial version of the KOOS-Child scale. Finally, a second meeting of the group of experts was held to compare and discuss the original and back-translated versions, resulting in the development of a pre-final version.

Subsequently, a pilot study was conducted on a sample of 10 children affected by knee problems to evaluate the clarity of this version, and necessary modifications were made to obtain the final Moroccan version.

Study population

After obtaining approval from the ethics committee, we conducted a study in the Pediatric Orthopedic service at Centre Hospitalier Universitaire (CHU) Hassan II, Fes, between September 2021 and June 2022. Two groups of participants were recruited: a group of children affected by knee problems and another group serving as a control, free of any knee pathology.

All children who spoke Arabic as their mother tongue and were aged between 6 and 16 years were invited to participate in the study, either in the group of patients or controls, depending on the presence or absence of a knee pathology diagnosed by an orthopedic surgeon.

Data collection

After obtaining consent from the child and their legal representative, participants were asked to complete a questionnaire. They could do so independently or with the assistance of their parents. The questionnaire included information on personal and clinical data, as well as various KOOS-Child scale questions. Where necessary, an interviewer was present to assist in completing the questionnaire.

Participants were asked to complete the KOOS-Child scale again after a minimum period of 15 days.

Statistical analysis

The five subscale scores of the Moroccan version of the KOOS-Child scale were calculated as the sum of the individual questions within each subscale. Raw scores were then transformed to a 0-100 for each subscale using the formula [100 −  (subscale mean score x 100)/4] [23]. A score of 0 represents extreme knee problems, and a score of 100 represents no knee problems. A global score was not calculated, as it is preferable to analyze and interpret the various subscales individually [23].

Initially, a descriptive analysis was used to describe the personal and clinical characteristics of the participants, along with the scores of the different subscales. Percentage values were used to present qualitative variables, while mean ± standard deviation (SD) was employed to present quantitative variables.

To ensure comparability between the group of patients and the group of controls, a comparison of baseline measures (age, sex, residence, and sports) was conducted. Percentages were compared using the Chi-square (χ^2^) test, and means were compared using the Student's t-test. A *P*-value ≤ 5% was considered significant.

The internal consistency of each subscale was assessed using Cronbach's alpha (α). A value greater than 0.9 was considered excellent, while a value less than 0.6 was considered poor [24].

The test-retest reproducibility of the KOOS-Child scale, between the first administration and the one 15 days later, was assessed by measuring the intraclass correlation coefficient (ICC) and its 95% confidence interval. ICC values less than 0.5, between 0.5 and 0.75, between 0.75 and 0.9, and greater than 0.90 successively indicate low, moderate, good, and excellent reliability [25].

To assess the discriminant validity of the KOOS-Child scale, a comparison of each subscale between the two groups (with and without knee injuries) was carried out. After testing the normality of the KOOS-Child subscales in its Moroccan version, the nonparametric Mann-Whitney U-test was used to compare the subscale distributions between the two groups. A *P*-value ≤ 5% was considered significant.

A confirmatory factor analysis (CFA) was conducted to evaluate whether the subscales of the Moroccan version of the KOOS-Child scale cluster in a manner consistent with the five subscales of the original version. For this, we used the Comparative Fit Index (CFI) and the Tucker-Lewis Index (TLI), with values >0.95 adopted as indicative of an acceptable model [26]. The standardized root-mean-square residual (SRMR) and the root-mean-square error of approximation (RMSEA) were also used, and a value <0.06 was considered indicative of a well-fitting model [26]. All indicators have values between 0 and 1, except TLI, which can be greater than 1 or slightly less than 0 [27].

The statistical analysis was performed using IBM SPSS Statistics for Windows, Version 26 (IBM Corp., Armonk, NY).

## Results

This study included 130 patients aged 9.82 ± 3.16 years, of which 82 (63.1%) were males and 77 (59.7%) lived in an urban area. The majority (115, 88.5%) did not practice sports activities.

A total of 88 patients and 42 controls were included. The baseline comparison revealed no statistically significant difference between the two groups (Table 1).

**Table 1:** Description and comparison of baseline characteristics between cases and controls.

		General characteristics, *n* (%)	Comparison of baseline characteristics between case and control groups		*P*-value
			Cases (88)	Controls (42)	
Age (mean ± standard deviation) in years		9.82 ± 3.16	9.6 ± 3.1	10.3 ± 3.2	0.267
Sex					
Girls		48 (36.9)	30 (34.1%)	18 (42.9%)	0.437
Boys		82 (63.1)	58 (65.9%)	24 (57.1%)	
Residence					
Rural		52 (40.3)	31 (35.2%)	21 (51.2%)	0.437
Urban		77 (59.7)	57 (64.8%)	20 (48.8%)	
Sports					
No		115 (88.5)	76 (86.4%)	39 (92.9%)	0.384
Yes		15 (11.5)	13 (13.6%)	3 (7.1%)	
Disease type					
Traumatic		-	30 (34.1%)	-	
Infectious		-	24 (27.3%)	-	
Malformative		-	26 (29.5%)	-	
Tumoral		-	4 (4.5%)	-	

Acceptability

The Moroccan version of the KOOS-Child scale had good acceptability, as no missing data or multiple responses were recorded. The time to complete the questionnaire varied between 9 and 13 minutes.

Table 2 presents the scores for various subscales of the Moroccan version of the KOOS-Child. The average scores ranged from 60.9 ± 39.9 for the sport subscale to 75.2 ± 33.4 for the pain subscale.

**Table 2:** Description and psychometric properties of KOOS-Child (internal validity and reliability [test-retest]). ADL, activities of daily living; KOOS, Knee Osteoarthritis Outcome Score; ICC, intraclass correlation coefficient; SD, standard deviation; CI, confidence interval

	Number of items	Mean score ± SD	Cronbach’s α	Reliability (test-retest) ICC (95% CI)
Symptoms	7	66.7 ± 14.7	0.47	0.85 (0.77-0.99)
Pain	8	75.2 ± 33.4	0.96	0.95 (0.93-0.97)
ADL	11	73.3 ± 34.3	0.99	0.98 (0.96-0.98)
Sports	7	60.9 ± 39.9	0.98	0.99 (0.98-0.99)
Quality of life	6	67.9 ± 33.9	0.97	0.99 (0.98-0.99)

Internal consistency

The details of Cronbach's α are presented in Table 2. All subscales, except for the symptom subscale, demonstrated excellent internal consistency (Cronbach's α > 0.9), while the symptom subscale exhibited lower consistency with a value of 0.47. However, the internal consistency of the symptom subscale improved after eliminating questions S4 (In the past 7 days, how many times were you able to straighten your knee completely by yourself?) and S5 (In the past 7 days, how many times have you been able to bend your knee completely by yourself?), resulting in Cronbach's α values of 0.706 and 0.646, respectively.

Reliability

Table 2 provides details of the ICC for all Moroccan KOOS-Child subscales. The values ranged from 0.85 (0.77-0.99) for the symptom subscale to 0.99 (0.98-0.99) for the sports and QoL subscale.

Discriminant validity

The discriminant validity is given in Table 3. Scores on all subscales of the Moroccan version of the KOOS-Child were statistically higher in controls than in cases (*P* = 0.003 for symptoms and *P* < 0.0001 for other subscales).

**Table 3:** Comparison of KOOS-Child subscale scores between cases and control groups. ADL, activities of daily living; KOOS, Knee Osteoarthritis Outcome Score; SD, standard deviation

Subscales	Cases (mean ± SD)	Controls (mean ± SD)	*P*-value
Symptoms	64.4 ± 17.1	71.7 ± 4.8	0.003
Pain	65.6 ± 34.7	95.5 ± 18.5	<0.0001
ADL	62.4 ± 35.0	96.1 ± 17.6	<0.0001
Sports	45.1 ± 36.6	94.3 ± 22.2	<0.0001
Quality of life	54.9 ± 32.1	95.1 ± 17.2	<0.0001

Among the cases, the lowest score was in the sports subscale (45.1 ± 36.6), while the highest score was in the pain subscale (65.6 ± 34.7). On the other hand, in controls, the lowest score was on the symptom subscale (71.7 ± 4.8), and the highest score was on the ADL subscale (96.1 ± 17.6).

Factor structure validity

The confirmatory factor analysis confirmed the validity of the factor structure of the Moroccan version of the KOOS-Child scale according to the structure of the original version, as all the indices were within the norms. The details are presented in Table 4.

**Table 4:** Goodness-of-fit indices for the KOOS-Child scale. CFI, comparative fit index; TLI, Tucker-Lewis index; SRMR, standardized root-mean-square residual; RMSEA, root-mean-square error of approximation

	CFI	TLI	SRMR	RMSEA (90% confidence interval)
KOOS-Child	1	1.03	0.003	0.001 (0-0.10)

## Discussion

The main objective of this study was to determine the internal consistency, reliability, discriminant capacity, and construct validity of the adapted Moroccan version of the KOOS-Child scale. The translation and cross-cultural adaptation process of the KOOS-Child into Moroccan Dialectal Arabic encountered no difficulties.

The Moroccan version of the KOOS-Child scale was found to be practical, reliable, and suitable for assessing problems that children and adolescents with knee disorders may encounter. It exhibited good test-retest reliability, as all ICCs were above 0.80 for all subscales. These findings are consistent with the validation results of the German and Egyptian versions of the scale [28,29].

The Moroccan version of the KOOS-Child scale demonstrated excellent internal consistency, as all Cronbach's α values were greater than 0.90, except for the symptoms subscale (α = 0.470). This finding is consistent with validation results from the German version of the KOOS-Child scale, which reported a Cronbach's α value of 0.6 for the symptom subscale at T1 and 0.52 at T2 [28]. Similar results were observed in the French Canadian version, where Cronbach's α values for the symptom subscale were 0.622 in cases and 0.572 in the control group [30]. 

In this study, the internal consistency of the symptoms subscale improved after removing questions S4 (In the past 7 days, how many times were you able to straighten your knee completely by yourself?) and S5 (In the past 7 days, how many times have you been able to bend your knee completely by yourself?), reaching values of 0.706 and 0.646, respectively. When both questions were removed simultaneously, the internal consistency increased further (α = 0.910). This suggests that the internal consistency of this subscale was affected by these specific questions, particularly as they were also responsible for decreasing the internal consistency of the symptoms subscale in the French Canadian version [30] and the German version [28]. The likely explanation is that some children misunderstood or misinterpreted the choices of these questions, which ranged from *never* to *always*.

It is worth noting that the homogeneity of the symptoms subscale was not sufficient during the validation of the original version of the KOOS-Child scale (α = 0.59), while it was good for all the other subscales (Cronbach's α = 0.80-0.90) [22]. These questions posed comprehension problems for the children during the initial development of the scale, as more than a quarter of the participants felt that questions P3 and P4 of the *pain *subscale, which focus on full knee flexion and extension, were duplicative of questions S4 and S5 of the symptom subscale, as they focused on the ability to perform the movement rather than on the pain during the movement [21].

In this study, the discriminant validity of the Moroccan version of the KOOS-Child scale was confirmed through the use of a control group, wherein the scores of the different subscales were statistically higher. This finding aligns with the results of the validation of the Canadian French version [30], which was the only study to employ a control group for discriminant validity assessment. In contrast, other validation studies of the scale confirmed concurrent validity by comparing it with other scales measuring the same dimensions [22,29,31].

Baseline scores of the KOOS-Child scale were established in healthy Australian children aged 8 to 17 years. The mean scores ranged from 92.9 for the pain subscale to 99.1 for the ADL subscale [32]. A similar range of scores was found in the French-Canadian validation control group, where the scores ranged between 93 and 96, except for the QoL subscale, where the score was 89. This difference was attributed to the fact that the control group was not entirely composed of healthy individuals [30]. In this study, all control group scores were above 94, except for the symptoms subscale score, which was 71.7. This discrepancy can be attributed to the challenges in understanding questions S4 and S5 of this subscale.

Thus, the confirmatory factor analysis indicated that the structure of the Moroccan version of the KOOS-Child scale was acceptable.

The main limitation of this study is the small sample size, which was mainly due to difficulties in recruiting patients. Despite this limitation, the questionnaire proved to be reliable and internally valid, except for the symptom subscale where questions S4 and S5 were poorly understood, leading to a decrease in the internal validity of this subscale. This issue needs to be addressed and retested in a revised version of the questionnaire.

Having a Moroccan version of the KOOS-Child scale enables us to compare the results of our studies with those of other research. It also facilitates the conduct of cost utility studies, where the use of a valid national scale is essential, as well as clinical trials that require such a scale to meet validity criteria.

## Conclusions

The results of the psychometric properties of the Moroccan Koos-Child scale showed that the Moroccan version had good acceptability, reliability, and discriminative capacity.

The internal consistency was good for all subscales, except for the symptoms subscale. A revision of questions S4 and S5 is required to improve the internal consistency of this subscale.
